# The Israeli health system’s rapid responses during the COVID-19 pandemic

**DOI:** 10.1186/s13584-024-00596-x

**Published:** 2024-03-04

**Authors:** Bruce Rosen, Michael Hartal, Ruth Waitzberg

**Affiliations:** 1 Myers-JDC-Brookdale Institute, JDC Hill, PO Box 3886, 91037 Jerusalem, Israel; 2 Hebrew University of Jerusalem, Jerusalem, Israel; 3 Technische Universität Berlin, Berlin, Germany

**Keywords:** COVID-19, Pandemic, Rapid response, Israel

## Abstract

**Background:**

The COVID-19 pandemic posed numerous challenges to health systems around the world. In addressing many of those challenges, Israel responded quite rapidly. While quick action is not an end in it itself, it can be important in responding to disease outbreaks. Some of Israel's rapid responses to the pandemic contributed significantly to population health and provided important learning opportunities for other countries.

**Main body:**

Some of the most prominent Israeli rapid responses were related to vaccination. Israel led the world in the pace of its initial vaccine rollout, and it was also the first country to approve and administer booster vaccines to broad segments of the population. In addition, Israeli scholars published a series of timely reports analyzing vaccination impact, which informed policy in Israel and other countries. Israel was a rapid responder in additional areas of public health. These include the partial closure of its borders, the adoption of physical distancing measures, the use of digital surveillance technology for contact tracing, the use of wastewater surveillance to monitor viral spread, and the use of vaccine certificates ("green passes") to facilitate a return to routine in the face of the ongoing pandemic. Many factors contributed to Israel's capacity to repeatedly respond rapidly to a broad array of COVID-19 challenges. These include a national health insurance system that promotes public–private coordination, a system of universal electronic health records, a high level of emergency preparedness, a culture of focusing on goal attainment, a culture of innovation, and the presence of a strong scientific community which is highly connected internationally. In addition, some of the rapid responses (e.g., the rapid initial vaccination rollout) facilitated rapid responses in related areas (e.g., the analysis of vaccination impact, the administration of boosters, and the adoption of green passes). While rapid response can contribute to population health and economic resilience, it can also entail costs, risks, and limitations. These include making decisions and acting before all the relevant information is available; deciding without sufficient consideration of the full range of possible effects, costs, and benefits; not providing enough opportunities for the involvement of relevant groups in the decision-making process; and depleting non-renewable resources.

**Conclusions:**

Based on our findings, we encourage leaders in the Israeli government to ensure that its emergency response system will continue to have the capacity to respond rapidly to large-scale challenges, whether of a military or civilian nature. At the same time, the emergency response systems should develop mechanisms to include more stakeholders in the fast-paced decision-making process and should improve communication with the public. In addition, they should put into place mechanisms for timely reconsideration, adjustment, and—when warranted—reversal of decisions which, while reasonable when reached, turn out to have been ill-advised in the light of subsequent developments and evidence. These mechanisms could potentially involve any or all branches of government, as well as the public, the press, and professional organizations. Our findings also have implications for health system leaders in other countries. The Israeli experience can help them identify key capacities to develop during non-emergency periods, thus positioning themselves to respond more rapidly in an emergency. Finally, health system leaders in other countries could monitor Israel's rapid responses to future global health emergencies and adopt selected actions in their own countries.

## Background

“Health system resilience” has been broadly defined as “institutions’ and health actors’ capacities to prepare for, recover from and absorb shocks, while maintaining core functions and serving the ongoing and acute care needs of their communities” [1–3]. While this definition focuses on health system functions, it recognizes the interrelations between a society’s health, social and economic needs, and associated wider systems [4, 5]. Recent literature has defined a variety of analytical frameworks to assess resilience specifically in the context of COVID-19, most of them relying on the WHO’s building blocks framework [6–11].

However, it is difficult to measure or assess “health system resilience”, in part because it is a dynamic and evolving process [4]. “Governance” is considered a core element in a health system resilience, yet it is one of the underexplored functions of health systems towards resilience [12]. In the context of the COVID-19 shock, it is argued that “timeliness and effectiveness of the government's response to COVID-19 had a great influence on the health system’s resilience” [11].

This review analyzes Israel’s rapid responses during the pandemic, as a key component of towards a resilient response to COVID-19. In line with Witter et al.’s (2023) reconceptualization, rather than assessing indicators of resilience, or the outcomes of the pandemic on population health, we opted to assess processes, decision-making, their background and implementation [5].

There is a large and growing literature comparing COVID-19 responses across countries [13–19]. The creation of this literature was facilitated by the establishment of several databases that tracked a broad range of relevant government policies over the course of the pandemic in many countries [20–22]. Studies have compared policies across a wide range of areas, including lockdowns, physical distancing, masking requirements, school closings, contact tracing, and vaccination. The policy dimensions analyzed include the choice of policy tools, their intensity, and their timing; the extent to which individual freedoms were constrained; the extent of inter-regional autonomy; and the extent of centralized decision-making within national government.

Studies have used a variety of conceptual models and approaches to explore possible causes of inter-country variation. The explanatory factors examined include type of government, government capacity and effectiveness, political party preferences, trust in government on the part of the public and professionals, partisanship/polarization, democratic principles and institutions, health system characteristics/capacities, tradition/commitments to individual liberties, economic strength, and budgetary health.

The databases of policy responses typically include data on the timing of the policy responses, and in some cases, it is possible to identify which countries responded most rapidly to particular challenges or were quick to institute particular measures. For example, Turkey, Italy, and the USA were the first OECD countries to announce selective border entry restrictions. Hong Kong, South Korea, and Switzerland imposed physical distancing measures such as restrictions to mass events more than a week before the declaration of a state of pandemic by the WHO [13]. Several small countries such as Malta were also fast in rolling out mass vaccinations [23]. Rwanda and Taiwan were rapid responders in many measures, including closure of schools, limitations to mass events and public life, and border closure [13].

A recent IJHPR article by Ginzburg et al. systematically compared mitigation policies implemented by Israel and their timing—*during the first wave of the pandemic*—to those of other OECD countries [13]. They found that Israel ranked in the upper third of OECD countries in swiftness of implementation for eight of the ten measures compared. Ginzburg et al. also made use of multivariate models to identify national characteristics associated with response rapidity. One of the key findings from that analysis was that "Countries with lower pre-pandemic socio-economic indices were quicker to initiate forced social distancing". They also found that "In Cox regression models, controlling for geographic location, democracy level above the OECD median was associated with a longer time-to-implementation of a lockdown".

While Ginsburg’s quantitative analysis is very valuable, it is important to understand the context in which the responses were implemented, and how to interpret these responses. For example, many liberal democratic countries are members of the EU which makes it more challenging to rapidly and legally undertake some of the responses such as limiting freedom of movement. Therefore, the relationship between the level of democratic society and time-to-implementation of a lockdown, should be taken cautiously.

While the Ginzburg et al. study is highly informative regarding the first phase of the pandemic, to date no studies have been published that consider the rapidity of Israeli responses to the pandemic throughout all its phases. Similarly, no studies to date have sought to identify a broad range of Israel-specific factors that may have contributed to the rapidity of the responses. The current review seeks to provide information and insights in both of those areas.

## Main text

### COVID-19 responses in Israel

Some elements of Israel's response to COVID-19 were widely considered laudable, and others—perhaps less so. Some commendable components include Israel's highly effective vaccination rollout [24] and its system for nation-wide monitoring of COVID-related hospitalizations, morbidity, and mortality [25]. However, other elements of the Israeli pandemic response have been more controversial, including the severity and duration of lockdowns [26], the duration of school closures [27], and the ways in which risks were communicated to the public [26, 28], the use of digital surveillance technology for contact tracing [29] and the Green Pass [30–32]. In this article we focus on one particular aspect of the Israeli response—its rapidity in responding to several key challenges and opportunities. “Appendix 1” provides context on how Israel compared with other countries on key COVID-19 health outcomes.

As detailed below, in facing the numerous public health challenges posed by the various phases of the COVID-19 pandemic to its health system, Israel responded relatively quickly to many, though not all, of those challenges. In any case, some of Israel's rapid responses both contributed significantly to population health within Israel and provided learning opportunities for other countries. As we will explore below, rapid responses in conditions of great uncertainty can generate important benefits, but they also can entail costs, and they often involve taking on risks.

Studies assessing health systems' responses and resilience to COVID-19 are usually clustered based on the WHO’s six building blocks framework for a systematic analysis: governance, workforce, financing, medical products, health information, and service delivery [33]. We adopt Haldane and colleagues’ extended framework (2021), which further adds three elements to the analytical framework: community engagement, public health functions, and collaboration across sectors [10].

The ten areas where Israel was a rapid responder that are highlighted in this review belong to these three elements. Most responses belong to public health functions: the initial vaccine rollout, the introduction of booster doses, border closures, physical distancing, wastewater surveillance and vaccine passports/Green passes. Digital surveillance and the rapid publication of large-scale studies of vaccine safety and effectiveness belong to public health functions together with health information systems. The creation of a national database for integrating data on COVID-19 related morbidity, mortality, hospitalizations, and vaccines is an outstanding example of a successful response related to health information systems. Finally, the establishment of task forces focused on helping at-risk population subgroups highlights the element of community engagement.

While the focus of this article is on those areas in which Israel was a rapid responder, it is important to note from the outset that many of the health system responses in Israel were similar in scope and timing to those of other high-income Western countries. These include responses related to the WHO’s building blocks’ such as rapidly increasing the capacity of physical and human resources [17, 34] to ensure the continuous provision of health services for COVID-19 and non-COVID-19 patients [35, 36] compensating health providers for income losses or extra expenses during the first waves of the pandemic [37]. Moreover, there were areas in which other countries responded more quickly and effectively than Israel. One striking example is the leadership provided by the United States in promoting the rapid development of several novel COVID-19 vaccines [38–40]. At the same time, the rapidity of Israel's response was remarkable regarding procuring vaccines and vaccinating the population, a variety of mitigation policies [13], and surveillance measures. We present ten such areas, starting with those that are vaccine-related, followed by those that are not vaccine-related.

## Rapid responses related to vaccines

### The initial vaccine rollout

Some of the most prominent of Israel's rapid responses were related to vaccination. Israel was one of the first countries to roll out and widely implement a full and comprehensive two-dose national vaccination program. Its initial vaccine rollout, which began on December 20, 2020, proceeded at a very quick pace [24]. As indicated in Table 1, by the end of December 2020, 16% of the Israeli population had already been vaccinated once, compared with 3% for the UK, 3% for the US, and 0% for Canada. By the end of February 2021, over half of Israel's population had been vaccinated at least once, well above the comparable percentages for the UK, the US, and Canada.^1^

**Table 1:** Percentage of population vaccinated by the end of selected months. *Source*: [41]

Country	December 2020	January 2021	February 2021
Israel	16	35	54
US	3	14	30
UK	3	8	15
Canada	0	2	4

An article published in this journal at the end of January 2021 identified and explored 12 factors that contributed to Israel's rapid vaccine rollout [24]. Some of these were long-standing characteristics of the State of Israel which are extrinsic to health care, such as having a well-developed infrastructure for implementing prompt responses to large-scale national emergencies [42]. Others were also long-standing, but health-system-specific, such as the organizational, IT and logistical capacities of Israel’s four non-profit health plans.^2^ A third set of factors were more recent and were specific to the COVID-19 vaccination effort. These included timely procurement of a large quantity of vaccines relative to Israel’s population size, and the formulation of simple, clear, and easily implementable criteria for vaccine eligibility and priority in the early phases of the distribution process [24]. Further information about each of these factors can be found in a January 2021 IJHPR article on the vaccine rollout [24] and in the footnotes to this paragraph.

### The introduction of booster doses

By the summer of 2021, Israel was no longer the world leader in first dose vaccination rates [43]. As of June 30, 2021, it had been surpassed by the United Arab Emirates, Canada, Chile, the UK, and Singapore, with Italy, Germany, and the United States not far behind. But on July 30, 2021, Israel again took a pioneering step when it became the first country in the world to approve and begin rolling out a “booster” (i.e., third) dose for its elderly population [44]. It is noteworthy that this took place more than 3 weeks before the US FDA officially issued an emergency use authorization for booster doses on August 25 [45]. Israel’s decision to proceed with boosters before US FDA approval contrasts with Israel's decision to wait until *after* FDA approval regarding the initial vaccination rollout at the end of 2020 [24].^3^ The decision to authorize booster vaccination was made in the context of having documented waning vaccine effectiveness during the pandemic wave fueled by the Delta variant.

### Rapid publication of large-scale studies of vaccine safety and effectiveness

The timely publication of scientific findings is an important component of the global response to a pandemic, particularly in cases where the pathogen is relatively new and unknown. If the pathogen is also dangerous and rapidly spreading, speed of publication becomes particularly important. The publication of scientific findings—whether in peer reviewed journals or on high quality preprint servers^4^—enables researchers and policymakers to better understand the pandemic and then build upon that understanding in subsequent actions.

Within 18 months of the FDA approval of the Pfizer BioNTech vaccine, Israeli researchers had published, in peer-reviewed journals, 80 "highly cited papers" (i.e., articles whose citation counts ranked in the top 1%) about the vaccine's real-world safety and effectiveness [46]. Most of these studies made rigorous use of detailed and comprehensive individual-level data available from Israel's health plans, hospitals, and Ministry of Health. Others were based on data aggregated by locality and/or age group and used natural experimental design to estimate vaccination impacts. Although Israel's population comprises less than 1% of the combined populations of all OECD countries, articles with Israeli authors accounted for 9% of all highly cited papers on the COVID-19 vaccines. Several of these were the first large-scale studies of emerging vaccine-related issues.

Moreover, Israel was recognized as an important source of early and valid data regarding the safety and effectiveness of both initial vaccinations and boosters. As a result, several of the pathbreaking Israeli studies contributed substantially to the development of vaccination policy in the US and elsewhere [47, 48].

The rapid vaccine rollout in Israel, the capability of Israeli researchers to perform studies of the effects of vaccination, and the accessibility of good information, all facilitated rapid publication of rigorous vaccination impact studies. The nation's four health plans all have electronic health records systems that together cover the entire population. They can be, and were, relatively easily harmonized and unified by the Ministry of Health.^5^ Another contributing factor was the rich collaboration networks that Israeli researchers had developed over the years with their colleagues in other countries. Further information on each of these factors can be found in a November 2022 IJHPR article entitled "The role of Israeli researchers in the scientific literature regarding COVID-19 vaccines" [46] and in the footnotes to this paragraph.

## Rapid responses not related to vaccines

Israel was also a rapid responder to COVID-19 in areas not directly related to vaccination. These included a tightly controlled closure of its borders to reduce the importation of the virus, physical distancing, wastewater surveillance, digital surveillance techniques, and the adoption of a green pass program.

### Border closures

Israel was among the first countries to impose selective entry restrictions to arrivals, such as arrivals from China [13]. Israel was also among the first western countries to close off its borders when the Omicron variant emerged at the end of 2021. Israel's capacity to quickly close its borders is due largely to having a single major international point of entry, Ben-Gurion Airport, with very limited cross-border ground traffic (due to Israel's unique geopolitical situation).

### Physical distancing

Israel was also among the first OECD countries to impose physical distancing restrictions such as cancelling mass events, limiting social gatherings, closing entertainment venues, and imposing a nationwide lockdown [13]. As noted by Ginzburg and colleagues, some of the characteristics that contributed to cross-national differences in response rapidity—such as relatively low levels of education and GDP—did not exist in Israel. On the other hand, they note that the rapidity with which physical distancing restrictions were adopted in Israel may have been related to Israel's relatively low level of health care resources^6^ (such as hospital beds, and particularly intensive care unit beds). Deficits in the infrastructure of the educational system (large and crowded classes and poor ventilation) also played a role [49].

Several analysts have argued that the Israel-specific political context also contributed significantly to the rapidity, and forcefulness, of Israeli responses in the first wave of the pandemic^7^ [50, 51]. They note that that at the time of the outbreak, the Prime Minister was under indictment for several alleged criminal actions. These analysts contend that the Prime Minister's vigorous responses to the pandemic were motivated not only by the need to protect population health, but also by the Prime Minister's desire to remain in office, increase his public standing, and use his position to his advantage in the judicial proceedings against him. This interpretation is disputed by many of his supporters.

### Wastewater surveillance

In March 2020, Israel rapidly employed wastewater surveillance as a tool in providing an early indication of viral spread [52]. In doing so, it reapplied the strategy implemented almost a decade earlier, when Israel experienced a globally unprecedented silent outbreak of wild poliovirus in 2013^8^ [53–55]. Wastewater surveillance, as an indicator of viral circulation in a community, can be an important complement to the testing of individuals, particularly in situations where many individuals are unaware of the need for testing (e.g., asymptomatic infection), when not enough individual tests are available, or when surveillance through traditional case tracing is no longer possible due to the overwhelming number of cases.^9^

### Digital surveillance

Israel was also a pioneer among western countries^10^ in the use of digital surveillance technology as part of its contact tracing efforts [29, 56]. Systems for both voluntary and non-voluntary digital surveillance were instituted as early as the first wave of the pandemic (March 2020). In doing so, Israel drew on its experience in counter-terrorism efforts [56]. In addition, with its goal-focused approach to reducing threats to population health, Israel quite quickly decided to first address population health protection and subsequently make adjustments to take into account personal privacy concerns. In contrast, many other countries chose to hold off or refrain from launching digital surveillance systems until they had a chance to deliberate extensively on the trade-offs between the benefit to population health and the infringement upon personal privacy. There is no consensus among policy experts on whether and when a deliberative approach is to be preferred to a rapid response approach. What is clear is that they reflect quite different policy development styles and that they can have significantly different implications for health and societal wellbeing.

Digital surveillance, as well as other rapid non-vaccine responses, were facilitated by the involvement of Israel's national intelligence agencies (NIAs). This was the first time that the NIAs were called upon to take actions focused on public health, unrelated to combatting terrorism [56]. In March 2020, COVID-19 was rapidly considered a mass disaster event, and a state of emergency was declared. The NIAs provided international data to recommend the prompt closure of borders in March 2020 and assisted in the procurement of hospital equipment including ventilators on the international market, at a time when many countries were competing for these goods [17, 56]. Israel’s NIAs provided techno-logistical support to non-voluntary epidemiological tracing, tracking, and monitoring. While these abilities and actions facilitated rapid responses with generally positive public health outcomes, they were accompanied by a lively public discourse regarding the extent to which they came at the expense of basic individual rights to freedom, movement, and privacy [57–59].

Significantly, Israel's Supreme Court played an important role in shaping the nature of Israel's digital surveillance efforts.^11^ Inter alia, it raised significant concerns about involving the General Security Services in non-voluntary digital tracing, in the absence of legislation specifically permitting such a practice. This development underscores the importance of post hoc mechanisms (including, but not limited to, Supreme Court oversight) for promoting reconsideration and fine tuning of rapid responses, even during national emergencies.

### Vaccine passports/green passes

Israel was among the first countries to introduce vaccine certificates and passports ("green passes"), which allowed the bearer entry to certain events and facilities which were closed to individuals who had not been vaccinated, unless they had a recent negative COVID-19 test result [31]. The plan for Israel's green pass program was announced before the national vaccination campaign started, and it was officially launched as soon as the entire adult population was eligible for the vaccine (February 2021). A key objective was to facilitate a return to a more sustainable way of life in the face of the ongoing pandemic. An additional effect of the green pass program was to create an incentive to get vaccinated. There is some debate about whether, and the extent to which, this was also an objective which motivated the introduction of the green passes. There has also been debate about the extent to which Israel's green pass program took into account considerations of equity in access to the vaccine [30–32, 60, 61].

The early introduction of the green pass program was made possible by Israel's having reached broad vaccination coverage at an early date. It also reflected Israel’s prioritization of population health protection over individual liberties when balancing the trade-offs between the two—in this particular situation, and perhaps more generally. In addition, it reflected a more general tendency in Israel's pandemic responses toward quick and efficient deliberations followed by rapid action, as opposed to extensive and comprehensive deliberations resulting in delayed action.

Israel was also among the first countries to include children in the green pass policy, even before the vaccines were approved for children. In addition, it was the first country to revoke green pass certification for individuals who did not receive a third vaccine dose within a stipulated time limit, unless they had a recent negative test result. This allowed for maintenance of the green pass policy of clear identification of vaccinated individuals and imposing on them fewer restrictions than on unvaccinated individuals. Thus, here, too, policymakers prioritized the protection of population health over considerations of certain individual liberties. At the same time, the green pass program helped the government shorten lockdowns and periods of tight restrictions on social gatherings.

### Establishment of task forces focused on helping at-risk population subgroups

During the first wave, Israeli authorities understood that communication and measures should be tailored to different cultural groups' needs and beliefs, to gain their trust and compliance [62]. In August 2020, the management of Israel’s national program for addressing COVID-19 was assigned to Magen Israel, a new organizational unit with Ministry of Health leadership. Magen Israel then quickly established a special task force charged with focusing on the population of ultra-Orthodox Jews and another special task force focused on the Israeli Arab population—two groups considered at high-risk and with unique socio-cultural needs. The two task forces were established in consultation with leaders of the relevant communities and included professionals from within those communities [63]. An additional task force—Magen Avot—focused on aged and disabled residents of long-term care facilities.

Throughout much of 2020, the main responsibilities of the task forces were to disrupt the chain of transmission by encouraging compliance with COVID-19 restrictions: physical distancing, the wearing of face masks, the appropriate use of testing, and adherence to isolation and quarantine directives. To that end, each task force developed close working relationships with leaders of their target population, analyzed the group-specific barriers to desired behaviors, and developed tailored strategies for promoting desired behaviors.

Toward the end of 2020, these task forces were also charged with promoting vaccine uptake. They did so through four main strategies: analysis of the reasons for slow vaccine uptake, partnership with community and religious leaders, tailored messaging, and easing access to vaccination sites.

### Creation of a national database for integrating data on COVID-19 related morbidity, mortality, hospitalizations, and vaccines

Shortly after the outbreak of the pandemic, Israel's Ministry of Health established a National COVID-19 Database. It drew individual-level information from all Israeli health plans, hospitals, and medical laboratories. From the start, it included reliable and consistently defined data on a broad range of outcomes, including confirmed COVID-19 infections, and admissions attributable to COVID-19. In January 2021, data were added on vaccinations. Every Israeli citizen has a unique identification number, and this facilitated data linkage across institutions. Anonymized versions of the database were made widely available to Israeli researchers, who were able to use it to track trends in COVID-19 incidence, COVID-19 resource use, and vaccinations. The database also played a vital role in identifying disparities among regions and in assessing vaccine safety and effectiveness. [46, 64]

## Discussion

In the findings section of this article, we highlighted ten areas in which Israel was a rapid responder to the challenges posed by the COVID-19 pandemic during the first 2 years of the pandemic. In the discussion, we relate our study to previous related studies, identify Israel-specific factors which may have contributed to the rapidity of its responses, discuss some of the costs and risks endemic to rapid response, and consider implications for policy in Israel and beyond.

### Relationship to previous studies

This article expands upon the work of Ginzburg et al. (and complements their work) in several ways: it relates to a broader set of responses (i.e., not only those related to mitigation), it considers a longer time period (encompassing additional pandemic phases), and it relies on non-statistical techniques in identifying both the areas in which Israel's responses were relatively rapid and in identifying factors likely to have contributed to the rapid responses.

The current review joins an extensive body of literature on how COVID-19 policy evolved over time in individual countries. These articles synthesize information across a broad range of policy measures (though without attention to response rapidity) and explore the factors underlying individual countries' policy responses. Single country studies of this type have been published regarding the US [65], the UK [66], Japan [67], Iran [68], and other countries. There is a need for additional studies which take an integrative look at Israel's response to the pandemic across its various phases and considering many different aspects of that response.

### Factors that contributed to Israel's capacity to respond rapidly to a broad range of challenges

During the COVID-19 pandemic, Israel was able to mount a rapid response to several health system challenges, with some of these related to vaccination and others unrelated to vaccination. Many factors contributed to Israel's capacity to be a rapid responder, including the presence of a strong scientific community which is highly connected internationally, a national health insurance system that promotes public–private coordination, a system of universal electronic health records, a high level of health emergency preparedness, a national culture of focusing on goal attainment, and a culture of innovation.

As noted earlier, Israel also has a tradition of prioritizing population protection and other public interests, over limitations on individual liberties and privacy. This is part of a wider commitment to social cohesion and solidarity in the face of crisis, with the wider social good taking a prominent place in prioritization in relation to considerations of individual utilities and liberties. A prime example of this can be seen in Israel’s successive responses to a series of poliovirus outbreaks that occurred starting in 1988. In response to the initial polio event, which was localized, and which preceded the COVID pandemic by some 22 years, Israel launched a rapid, emergency nationwide vaccination campaign. This aggressive national response was met by observers with reservations with regard to its justification, as the limited scope of the local outbreak, its slow dynamics, and the high vaccine coverage in the national population could have allowed a more moderate and measured response. However, the decision to proceed with a vigorous, nationwide response was ultimately influenced heavily by social, cultural, and political factors, which favored this option over a more limited, localized or individualized response [69].

Another nationwide vaccination response was initiated in 2013 in response to the environmental detection of wild poliovirus in the sewer in southern Israel. Unlike the 1988 outbreak, there were no clinical cases identified in 2013, yet the response was, again, a national campaign to administer oral, live-attenuated polio vaccine. Notably, even subsequent to the COVID pandemic, and 35 years after the 1988 event, this approach has continued in response to additional localized polio outbreaks in 2022 and 2023, despite considerations of public vaccine hesitancy and high vaccine coverage rates [70, 71].

In addition, some of the rapid responses (e.g., the efficient initial vaccination rollout) positioned Israel to also respond swiftly in related areas (e.g., analysis of vaccination impact, administration of boosters, and the adoption of green passes).

The quick responses during COVID were also based in part on Israel's overall high level of emergency preparedness of Israel for varied adversities (i.e., not limited to health adversities). Israel has an “emergency culture” that allows for centralization of data, good "control and command’ operations, wide collaborations between authorities and responders, and routine cooperation between military and civilian systems [42]. Together, these facilitate rapid and relatively smooth responses to emerging and acute threats such as the COVID-19 pandemic.

Another contributing factor was the ability to expand capacities by accessing resources routinely allocated for other purposes. For example, Israel's national emergency medical service (Magen David Adom) was used to conduct COVID-19 testing for home-bound patients, and to assist in the administration of vaccines. Similarly, military personnel and intelligence services were called upon to assist with contact tracing, and the military also played an important role in the national vaccination campaign.

A rapid response requires both the capacity to respond rapidly and a willingness to do so. It appears that both capacity and willingness to apply that capacity have contributed to the volume and range of Israel's rapid responses to COVID-19.

### Costs, risks, and limitations associated with rapid responses

Was Israel’s rapid response beneficial? Although there may not be a single, simple answer to this question, the empiric data seem to suggest that, in terms of mortality, Israel’s handling of the pandemic response positioned it favorably relative to other comparable, developed, western countries. When comparing end-of-year cumulative confirmed COVID-19 deaths per 1 million population for the years 2020–2022, Israel’s rates, year after year, were similar to those of Denmark and Canada, and significantly lower than those of France, Germany, the UK, and the U.S. [72] Further information on how Israel compared with high-income countries on key COVID-19 outcome measures can be found in “Appendix 1”.

While rapid response clearly has many advantages, including but not limited to reduced population mortality, it can also entail costs, risks, and limitations. These include making decisions and acting before all the relevant information is available; deciding without sufficient consideration of the full range of possible effects, costs, and benefits; not providing enough opportunities for the involvement of relevant groups in the decision-making process (such as occurred with digital surveillance); and depleting non-renewable resources. We do not have evidence that these, in fact, manifested as major issues in the Israeli COVID-19 response, but they should be acknowledged and considered in the wider analysis of costs and benefits of rapid response.

There is a very broad consensus about the wisdom and efficacy of Israel's rapid vaccine rollout as it clearly contributed substantially to population health. In retrospect, it is also widely (though not universally) agreed that Israel chose wisely in launching the world's first booster drive—even before FDA approval—in the face of waning immunity and the Omicron wave. At the same time, it is important to note that before the decision was made to launch Israel's booster drive, there was uncertainty about the extent of its likely contribution to pandemic control, along with differences of opinion on whether it would be judicious to wait for an FDA decision. And while the boosters did make an important contribution to population health, their uptake was less widespread than for the primary doses.

Retrospectively, many experts question whether it was appropriate to rapidly adopt non-voluntary digital surveillance and strict borders closures [42]. In addition, as a pioneer in implementing vaccine certificates, Israel could also have been a pioneer in promoting public discussion of questions raised by the green pass such as its potential negative effects on equity, solidarity, and trust building [32, 61].

Furthermore, while the rapid implementation of vaccinations had a large positive effect on people’s health with relatively little monetary or social cost, the rapid implementation of some of the non-pharmacological interventions (such as cancellation of mass events, limiting social gatherings, a nationwide lockdown, and perhaps most significantly prolonged school closings) entailed much higher costs, economically, psychologically, socially, and in terms of impacts on democracy and civil rights [73–77].

Moreover, across a very broad range of decisions that faced policymakers during the pandemic, there were at least some Israeli experts who advocated policies different from those recommended by the government's official panel of expert advisors [26]. In some cases, this was because they differed from the official panel of experts regarding the extent to which the measure under consideration was likely to contribute to pandemic control. In other cases, they differed from the official panel of experts regarding the likely effect on societal considerations other than pandemic control.

Given the pandemic's enormous toll on population health prior to the introduction of vaccines, along with uncertainties at that time about whether/when effective vaccines would become available and how variants of the virus would evolve, a reasonable case can be made that these interventions and the rapidity with which they were implemented were appropriate at that time. On the other hand, perhaps a slightly less rapid, more deliberative decision-making process that had taken more time to weigh the various considerations could have resulted in a more optimal balancing of costs and benefits [78]. A slightly slower pace might also have provided more time for public engagement in the decision-making process, leading to broader acceptance and implementation.

Moreover, as more data become available it is important to revisit policy decisions and to do so promptly. This may be particularly important in cases where policymakers make the decisions quickly. In the case of school closures, it took Israel longer than many other countries to revisit that decision and ultimately put an end to the closures.

Further information on the benefits and costs of each rapid response can be found in “Appendix 2”.

A resilient response to a shock to the health system like COVID-19 entails not only rapid responses, but also being prepared for such shocks. Preparedness does not only mean having excess capacity and resources, as some frameworks suggest [7–9, 11], as these can represent difficult tradeoffs with efficiency, particularly in a stretched health system such as the Israeli one [79]. Preparedness also means having response plans in place, with a coordinated governance that enables following these plans. A resilient system has a functioning health system in routine times, with as few disparities as possible [5]. The Israeli health system struggles to reduce disparities in routine times and should also do that during shocks. We encourage researchers to analyze the outcomes of Israel’s rapid responses beyond the health outcomes typically assessed, and investigate disparities in health outcomes, and other socio-economic aspects.

Rapid responses can also have important limitations. For example, rapidity of decision making does not ensure full and equal implementation.^12^ While Israel was quick to impose physical distancing requirements, these were not well enforced in the ultra-Orthodox sector, due to a variety of cultural and political considerations. In addition, rapidity of decision making, and rapidity of implementation, do not ensure persistence and goal achievement. While Israel was an early leader in vaccination rates, it subsequently fell behind many other countries.^13^

To sum up, the ability and willingness to respond rapidly often brings with it many advantages (e.g., reduced morbidity and mortality), but it can also bring with it disadvantages in terms of insufficient consideration of the broad range of potential effects, insufficient involvement of some elements of society, and decisions which prove to have been unwise once additional information comes to the fore.

## Conclusions

In the foreseeable future, rapid response will, and should, continue to be a key component of Israel's cultural and societal DNA for dealing with national emergencies, including health emergencies. Looking to the future, Israel can improve upon its COVID-19 experience by engaging a broader set of actors in fast-paced decisionmaking processes and improving communication with the public. Furthermore, public sector agencies can use the current inter-pandemic phase to learn, adopt and integrate principles of organizational agility [80], which can be expected to improve mechanisms for promptly reconsidering, adjusting, and even reversing decisions which, while justified at the time that they were made, are no longer appropriate in light of subsequent evidence.

Israeli policymakers must also revalidate the balance between population health protection, individual liberties, and privacy; between physical and mental health; and between various health and economic considerations in emergency scenarios. These tradeoffs will continue to be central issues as technology develops, data become more available, real-time data analysis becomes more feasible, and social media continue to erode personal privacy. The trade-offs should be carefully considered and transparently deliberated, with the involvement of a variety of stakeholders, including the Ministry of Health, the Prime Minister's Office, the Israel National Digital Agency, the NIAs, health care providers, the public, and Israel's parliament (the Knesset). Doing so could be important for ensuring societal acceptance of stringent policies when they are most needed.

Israel's National Institute for Health Policy should play a leadership role in the next phase of this process by convening the key actors to discuss, in a non-pressured and neutral environment, the pros and cons of rapid response during the COVID-19 pandemic and the lessons for future national emergencies. The National Institute could also fund a survey of population preferences regarding the tradeoffs involved in rapid responses, as well as an analysis of how the survey findings on public preferences should be factored into policy development [78]. The National Institute is well suited to provide leadership on these activities as it has extensive experience as a neutral convener of policy development forums, a research funder, and a translator of research findings into policy recommendations. As the issues go beyond health care, the National Institute might want to partner in this effort with a think tank or similar organization with expertise in public policy development. The results of this consultative process would be forwarded to the appropriate Knesset committees for their consideration and action.

The present review also has implications for health system leaders in other countries. The Israeli experience can help them identify which capacities they might wish to develop during non-emergency periods that would enable them to respond more rapidly to national health emergencies. For example, U.S. experts with whom we have shared the Israeli experience, have expressed interest in learning from Israel about how it quickly develops new national databases in response to national emergencies. In addition, this article suggests that, during periods of global health emergencies, health system leaders in other countries could benefit from monitoring Israel's rapid responses and considering which of them to implement in their own countries.^14^

No single country demonstrated perfect performance during the pandemic, and many health care systems, including Israel's, have ample room for improvement in advance of future national and global health crises. By comparing and contrasting experiences and lessons learned, health system leaders can more clearly identify areas of relative strengths and weaknesses within their own systems and consider methods to introduce improvements informed by the accomplishments—and the blunders—of others.

By systematically analyzing Israel's areas of rapid pandemic response and elucidating their root causes, we have provided a framework to foster this type of international learning. Furthermore, we believe that this analysis can also facilitate systematic improvement within Israel, specifically in revalidating the balance between population protection and personal liberties, and in adopting agile organizational practices, which could make it easier to review new evidence, incorporate it into decision making processes, and update, cancel, or even reverse previous decisions as it becomes clear that they are no longer congruent with the emerging evidence.

## Data Availability

Not applicable.

## Appendix 1

### A brief overview of how Israel compared with the average of all high-income countries on key COVID-19 health outcomes

Our World in Data (OWID) has published the following statistics [72], which aggregate data over the full course of the pandemic (February 2020 to May 2023):

- The rate of COVID-19 confirmed deaths per thousand was lower in Israel than the average for all high-income countries (1.3 v. 2.3)
- The rate of COVID-19 confirmed cases per thousand was higher in Israel than the average for all high-income countries (510 v. 337)
- The case fatality rate was much lower in Israel than the average for all high-income countries (0.26% v. 0.68%)

The use and interpretation of these data should be approached with caution as they are complicated by potential differences across high-income countries (HICs) in the age mix (with Israel having a relatively young population), the rates of case finding (due in part to differences in the availability of tests), and the procedure for attributing deaths to COVID-19.

OWID data on excess mortality rates (EMR) are less subject to those concerns, but OWID does not publish a mean EMR statistic across HICs. OWID's country-specific data indicate that Israel's EMR over the full pandemic period (8%) was lower than those of the US (14%) and the UK (10%), but higher than those of Germany and France (both 6%). However, even data on EMR must be approached and interpreted cautiously due to contextual and methodological differences between countries.

Further studies are needed to explore the meaning of these more fully and related statistics. In addition, future studies should examine how Israel's relative position on key outcome variables changed over the course of the pandemic, and how these outcome differences are related to differences across countries in how they responded to COVID-19.

## Appendix 2

### The rapid responses: a retrospective assessment

As indicated in Table

**Table 2:** Retrospective assessments of each of the ten rapid responses

Rapid response	Contribution to pandemic control	Negative impacts
1 The initial vaccine rollout	Yes [81]	Limited: autonomy; side effects
2 The introduction of booster doses	Yes [82]	Limited: autonomy; side effects
3 Rapid publication of large-scale studies	NA	None
4 Border closures	Yes [83]	Moderate: autonomy; economic loses
5 Physical distancing	Yes [83]	Substantial negative impact on the economy and educational processes; some negative impact on physical and mental health
6 Wastewater surveillance	NA	None
7 Digital surveillance	Yes [84]	Moderate to substantial: intrusion into personal privacy in case of involuntary surveillance; dangerous precedent beyond health care
8 Vaccine passports/green passes	Yes [85, 86]	Moderate: limits on autonomy including freedom of movement and assembly
9 Task forces on at-risk subgroups	NA	None
10 Integrated national database	NA	None

2, subsequent evidence has demonstrated that most of the types of interventions that were rapidly adopted by Israel have indeed contributed substantially to containing the spread of COVID-19 and/or its more severe health effects. These include initial vaccine doses, booster doses, border closures, physical distancing, digital surveillance, and vaccine passports.^15^

Nonetheless, there were, and continue to be, strong differences of opinion regarding the duration, the desired level of stringency, and the associated costs and disadvantages of some of these measures. Some of these are measures which, alongside their positive contributions to pandemic control also had significant negative impacts on other life domains, such as education, the economy and/or mental health or negative impacts on values such as personal freedom and privacy. People can differ on how they view the tradeoffs between health and other values/life domains. In addition, on some measures (e.g. school closures), the differences in assessments of appropriateness are primarily about the intensity and duration of the application of the measure, rather than whether the measure per se should have been adopted at all [56].

Interestingly, some of the responses we studied are among the traditional public health responses to pandemics; these include vaccinations, border closures, and physical distancing. Others are newer and Israel was among the pioneers; these include vaccine passports and involuntary digital surveillance. With regard to the former, differences among experts related primarily to duration and stringency; with regard to the latter, they also related to whether to undertake the response altogether [29, 56, 61].

The upshot of all of this is that several of the interventions that Israel adopted rapidly are of the sort that require calibration as well as tradeoffs among competing values, and of the sort that deserve periodic reconsideration. As indicated in our conclusion section, we believe that decision-making frameworks and processes can be developed that support those objectives without unduly delaying responses when a rapid response is needed.

## Abbreviations

IJHPR: Israel Journal of Health Policy Research
MDA: Magen David Adom (Israel's national emergency medical service)
OECD: The Organization for Economic Cooperation and Development

## Footnotes

1 The Israeli rollout was particularly rapid and effective among the elderly. Among persons aged 60 and over, the percentage who had received at least one dose increased from 0 to 70% during the first 3 weeks of eligibility, while it took another 3 weeks to reach 80% and a further 4 weeks to reach 90% [63]. In contrast, in the United States it took 23 weeks until 80% of the populated aged 65 and over had been vaccinated at least once [87].

2 As elaborated under "Factor 4" in a 2021 article on Israel's vaccine rollout [24], the health plans were able to contribute to the speedy and efficient roll-out of vaccines due to their "well-developed electronic health records and strong capacities for electronic communications with their members", their "substantial experience in organizing and implementing nation-wide initiatives, large-scale mobilizations and emergency responses of various sorts", their experience with annual influenza campaigns, and other factors. These capacities and experiences facilitated the health plans' abilities to staff and "rapidly rent appropriate spaces for a mass vaccination drive, and to quickly distribute the vaccine to over 400 delivery points while meeting the challenging temperature and other logistic requirements of the Pfizer BioNTech vaccine". The health plans were also able to "quickly and efficiently schedule hundreds of thousands of vaccination appointments for their members via call centers, apps, and organizational websites.".

3 Factors that contributed to Israel's willingness to precede the FDA regarding booster doses—but not regarding the primary does—included the following: (a) Boosters involved fewer unknowns and lesser risks regarding safety and efficacy; (b) Israeli regulations require FDA or EMA approval with regard to use of new vaccines but not with regard to booster doses of existing vaccines; (c) Israel had acquired experience administering booster doses, and monitoring their effects, among its immune suppressed population; (d) By the time of the booster decision, Israeli policymakers has become very familiar with the real world studies of the impact of initial doses; (e) Israel understood that there was waning immunity sooner than the US and Europe due to the rapidity with which Israel had rolled out the initial vaccination campaign and close surveillance of new cases; (f) A perception that it would take time for US and European regulators to become aware of, and confident in, the early data on waning effectiveness, much of which came from Israel [88].

4 During the COVID-19 pandemic the world of scientific publishing changed in several important ways. These included the acceleration of review processes at peer-reviewed journals and a major increase in the prominence of pre-print servers. While these changes clearly enabled more rapid dissemination of findings, questions have emerged regarding the extent of the negative impacts on rigor and accuracy.

5 The Ministry of Health' National COVID-19 Database was set up rapidly specifically for COVID-19 pandemic management, drawing information from all Israeli health plans, hospitals and medical laboratories. It included reliable and consistently defined data on both vaccinations and a broad range of outcomes (including confirmed COVID-19 infections, and admissions attributable to COVID-19) [46].

6 Many of these factors also applied in other countries that were quick to institute physical distancing requirements, including several Mediterranean countries [17].

7 This relates to both physical distancing (item 5) and border closures (item 4).

8 At that time Israel was also a rapid responder, “inventing the wheel”, as there were no similar experiences with such an event [55]. During the COVID-19 pandemic, Israel re-applied this novel approach in new circumstances.

9 Ultimately, wastewater surveillance did not make a major public health or clinical contribution to control of the COIVD-19 pandemic, in contrast to the major contribution made in the case of the silent polio outbreak. This difference is probably attributable to the more rapid emergence of clinical symptoms, and greater availability of individual testing, in the COVID-19 pandemic.

10 Several Asian countries, including China, Singapore, South Korea, and Taiwan, were also quick to adopt digital surveillance techniques [29].

11 The Supreme Court’s involvement apparently contributed to the cessation of digital surveillance around July 2020.

12 Admittedly, the same can also be said of slow decisionmaking; it too does not ensure full and equal implementation.

13 This may be part of a broader phenomenon, that goes beyond public health. For example, Israel is better at creating high tech start-up companies than it is at scaling up and expanding them into very large corporations [57]. It may be that due to living in a small country, with significant security challenges and little strategic depth, Israelis may have become better at sprints than at marathons.

14 For some countries, this would be a natural continuation of practices adopted during the COVID-19 pandemic, in which Israeli vaccine policies and studies were closely monitored. In addition, during the pandemic a broad range of Israeli public health measures were monitored by many European countries through the reports of the European Observatory on Health Systems and Policies.
